# Through the Eyes: A Case of Ocular Syphilis

**DOI:** 10.7759/cureus.48236

**Published:** 2023-11-03

**Authors:** Samuel Nwaobi, Amaka C Ugoh, Blessing C Iheme, Agatha O Osadolor, Rashid K Walker

**Affiliations:** 1 Family Medicine, Piedmont Columbus Regional-Midtown, Columbus, USA; 2 Family Medicine, University of Benin Teaching Hospital, Benin, NGA; 3 Family Medicine, American University of Barbados, Bridgetown, BRB; 4 Public Health Sciences, Xavier University School of Medicine, Miami, USA

**Keywords:** ocular syphilis, infectious venereal disease, neurosyphilis, treponema pallidum, syphilis, spirochetes

## Abstract

Syphilis is a bacterial disease caused by Treponema pallidum and is sexually transmitted via vaginal, anogenital, or orogenital contact. Vertical transmission between mother and unborn child is also possible, but transmission via skin-to-skin or blood contact is rare. The objective of this case is to highlight this rare ocular manifestation of syphilis as it manifests as a multisystemic disease affecting many organ systems. This is a case of a 46-year-old male with vision loss who was referred to the emergency department by an ophthalmologist. Two days before the presentation at the emergency department, the ophthalmologist observed the presence of optic disc edema in the left eye. At the emergency department, he complained of bright light spots in the left eye and complete darkness in the central aspect of the eyes. He reported having a non-pruritic erythematous rash on the anterior abdomen that began one week before his presentation at the emergency department. The patient also reported having multiple sexual partners. Physical examination findings showed a visual field defect in the left eye, normal bilateral eye movement, and a non-tender skin reticulation over the anterior abdominal wall. Lab results showed complete blood count (CBC) and comprehensive metabolic panel (CMP) within normal limits,* *fluorescent treponemal antibody (FTA) antibody reactive, human immunodeficiency virus (HIV) test and hepatitis panel negative, rapid plasma reagin (RPR) titer 1:64, and imaging results negative for any significant abnormalities. The infectious disease specialists were consulted, and the recommended IV penicillin of four million units every four hours was given. The patient reported an improvement in his blurry vision over three days. By six months, his vision was back to baseline. This case report is significant due to the rare occurrence of ocular complications as an initial presentation of syphilis. Considering the rising cases of syphilis in the United States, it is important to highlight the possibility of this uncommon clinical presentation of syphilis.

## Introduction

Syphilis is a sexually transmitted disease caused by the spirochete Treponema pallidum. T. pallidum spirochetes may be detected directly using histopathologic staining, darkfield microscopy, direct fluorescent antibody, and polymerase chain reaction (PCR) assays. However, these are not widely available in hospitals; therefore, serologic testing is often used in diagnosing syphilis [1]. Syphilis is primarily spread through sexual contact with those who have active contagious sores. However, it may be transmitted by blood transfusions contaminated with the bacterium, from an infected mother to her fetus during pregnancy, and rarely through direct skin-to-infectious-lesion contact. Syphilis can progress through four separate phases if untreated (primary, secondary, latent, and tertiary). As a result of T. pallidum invasion, the characteristic primary syphilis presentation is a single, non-tender genital chancre. Patients may experience regional lymph node enlargement and multiple non-genital chancres, including those on their digits, nipples, tonsils, and oral mucosa. These original lesions often disappear without treatment, making early diagnosis difficult. Primary syphilis can develop into secondary and tertiary syphilis and, if left untreated, has a wide range of clinical presentations, ranging from cardiac, neuro-ophthalmological, and dermatological features [2]. According to the CDC, reported cases of sexually transmitted infections (STIs), such as chlamydia, gonorrhea, and syphilis, increased between 2020 and 2021, reaching more than 2.5 million reported cases. The CDC report also highlighted a 32% increase in syphilis for combined stages of the infection [3]. With the rising syphilis cases in the United States, there has been a corresponding increase in ocular syphilis [4,5]. A delay in the treatment of ocular syphilis may lead to vision loss. This case report presents a case of ocular syphilis as the initial presenting feature in an immunocompetent patient.

## Case presentation

**Figure 1:**
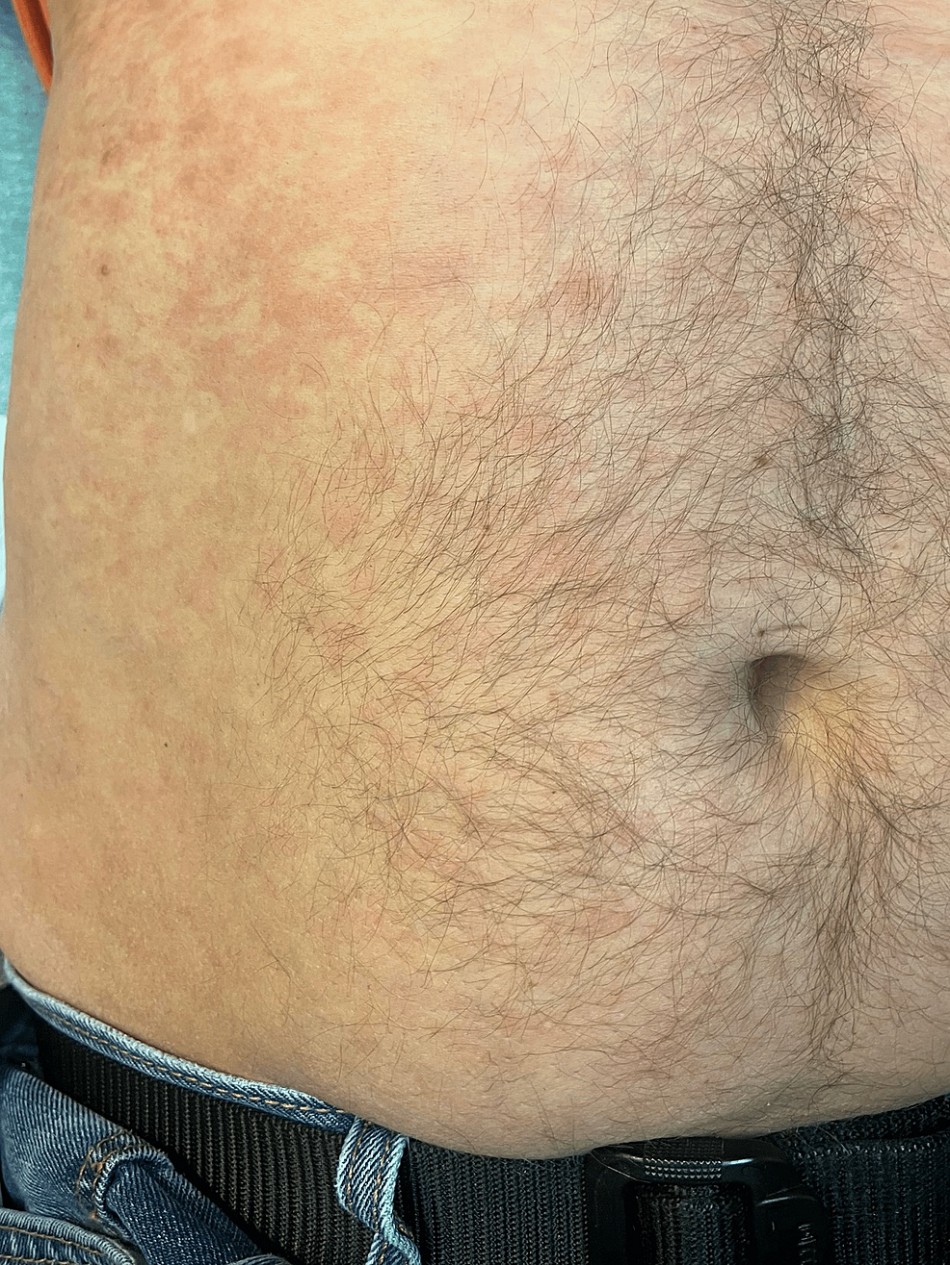
Non-tender Abdominal Rash

The patient is a 46-year-old male who presented to the Emergency Department (ED) because of progressively worsening vision changes in his left eye. About four weeks before his presentation, he began experiencing bright light spots in his left eye. His symptoms gradually worsened, and two days before this ED encounter, he noticed complete darkness in the central aspect of his left eye, which he reported to his ophthalmologist. On examination of the patient, the ophthalmologist had concerns about optic disc edema in the left eye and referred the patient to the ED for further workup. The patient reported noticing an erythematous truncal rash over his anterior abdomen on further history-taking. He stated he noticed the rash about a week before this presentation. The patient denied any fever, chills, or abdominal pain. He denied any recent illness or sick contacts. His medical history was otherwise significant for one pack per day of tobacco smoking, cocaine, and opioid use disorder and has been in remission for the past 10 years. He reported having multiple sex partners and unprotected intercourse during that period but has been in a monogamous relationship for the past five years. The patient denied any other chronic illnesses. His blood pressure was 115/75, his heart rate was 86 beats per minute, and the patient was afebrile. Physical examination showed a non-tender skin reticulation over the anterior abdominal wall (Figure 1). The patient had normal bilateral extraocular eye movements, a visual acuity of 20/40 in the left eye, and 20/30 in the right eye on the Snellen chart. There were no neurologic deficits on cranial nerve II-XII examination.

His complete blood count (CBC) and comprehensive metabolic panel (CMP) were within normal limits at the ED. To rule out intracranial abnormalities, such as a cerebrovascular accident, cerebral venous thrombosis, and cerebral abscess, the patient had a computed tomography (CT) of the head without contrast, magnetic resonance imaging (MRI) of the brain with and without contrast, and magnetic resonance imaging venogram of the head with contrast. These tests were negative for any acute abnormalities. Considering the patient's past social history, a syphilis screen, hepatitis panel, gonorrhea, chlamydia, and HIV testing were ordered. His RPR titer was noted to be 1:64, and FTA antibodies were reactive. The hepatitis panel, HIV, gonorrhea, and chlamydia tests were negative. A diagnosis of ocular syphilis was made, and an infectious disease specialist was consulted. The patient was started on IV penicillin of four million units every four hours. His truncal skin changes were presumed to be a case of livedo reticularis from syphilis infection. The patient was admitted for a 72-hour stay, and his overall health was monitored. There was no worsening of symptoms during hospitalization; rather, the patient did report improvement in his vision. He was discharged on intramuscular procaine penicillin 2.4 million units and probenecid 500 mg orally every six hours for 14 days. Follow-up care with ophthalmology and the health department was scheduled. The medical team discussed with the patient the need for partner testing and treatment. Six months after treatment, the patient had a repeat RPR titer of 1:2, and his vision was back to baseline. He denied any adverse reaction to his syphilis treatment.

## Discussion

Syphilis occurs in four stages, and these include the primary, secondary, latent, and tertiary stages. The pathophysiology of syphilis begins with the injection of the T. pallidum organism at the primary site of infection, and this manifests with chancre and regional lymphadenopathy. If left untreated, this can progress to secondary syphilis where such a patient is seen to manifest with a maculopapular or pustular rash involving the palms of the hands and the soles of the feet. The last stage, tertiary syphilis, involves the manifestations of gummas and neurological symptoms [6].

Although rare, ocular syphilis is one of the complications of T. pallidum infections and can occur at any stage of the disease. Previous studies have reported the incidence of syphilitic uveitis as ranging from 1% to 4% [7]. Its symptoms range from pain to blurry vision and even vision loss with posterior uveitis and pan-uveitis being the most common presentations [8]. The case definition for ocular syphilis is a person with clinical symptoms or signs of ocular disease with syphilis of any stage [9,10,11]. Ocular syphilis presents in several ways, including optic disc edema (as seen in the index patient), uveitis, retinitis, and vitreous and retinal vasculitis [12].

A high index of suspicion is required to make the diagnosis, and affected individuals must have serologic confirmation of syphilis while ruling out other causes of vision loss. In this patient, radiological imaging of the brain and blood vessels was done to rule out intracranial and vascular causes of vision loss, such as tumors and cerebral venous sinus thrombosis. Positive syphilis results should necessitate screening for immunosuppression (HIV) and other STDs to guide appropriate treatment plans. It is important to thoroughly examine all persons with reactive serology who have presented with ocular symptoms. Ideally, an ocular examination including a full cranial nerve examination should be completed. In general, if ocular abnormalities exist on physical examination in patients with reactive syphilis serology with no cranial nerve dysfunction or other neurologic abnormalities, CSF analysis is not necessary before the commencement of treatment. However, CSF analysis is still helpful for evaluating people with ocular symptoms and reactive syphilis serology, but negative ocular findings on examination do not rule it out [13].

The treatment of ocular syphilis is the same as that of neurosyphilis. Aqueous crystalline penicillin G 18-24 million units per day is recommended. It should be administered as three to four million units intravenously every four hours or as a continuous intravenous infusion for 10-14 days. An alternative regimen is procaine penicillin G 2.4 million units intramuscularly once daily, plus probenecid 500 mg orally four times daily, for 10-14 days [14]. Ocular syphilis can affect individuals irrespective of immunity status. Still, it has been noted that those with a low CD4 count and positive HIV status are more likely to progress faster to ocular manifestations of syphilis. The treatment modality for asymptomatic neurosyphilis regardless of the HIV status is benzathine penicillin G 2.4 million units, given intramuscularly weekly for three weeks. However, a slower serologic response to treatment is often expected in patients with neurosyphilis [15].

Syphilis progresses very rapidly; therefore, clinicians should be aware of ocular syphilis and screen for visual complaints in patients at risk for syphilis, such as persons with multiple sexual partners, men who have sex with men, and HIV-infected persons [16]. Safe sex practices, such as consistent condom use during intercourse, have been shown to decrease the likelihood of sexually transmitted infections such as syphilis [17]. In addition, if the disease is recognized and treated early, immunocompetent patients will have a better chance of recovery without long-term sequelae.

## Conclusions

There has been an increase in cases of syphilis in the United States, and this is gradually becoming a global challenge. Ocular syphilis is a rare manifestation that can occur either at the primary, secondary, or tertiary stages with presentations of either posterior uveitis or panuveitis. It is important to diagnose such patients promptly so that effective treatment can be administered to avoid permanent vision loss.
